# Insights from modeling into structure, entanglements, and dynamics in attractive polymer nanocomposites†

**DOI:** 10.1039/d1sm00683e

**Published:** 2021-06-09

**Authors:** Ahmad Moghimikheirabadi, Martin Kröger, Argyrios V. Karatrantos

**Affiliations:** Department of Materials, Polymer Physics, ETH Zurich, Leopold-Ruzicka-Weg 4 CH-8093 Zurich Switzerland ahmadm@mat.ethz.ch mk@mat.ethz.ch; Materials Research and Technology, Luxembourg Institute of Science and Technology 5, Avenue des Hauts-Fourneaux L-4362 Esch-sur-Alzette Luxembourg argyrios.karatrantos@list.lu

## Abstract

Conformations, entanglements and dynamics in attractive polymer nanocomposites are investigated in this work by means of coarse-grained molecular dynamics simulation, for both weak and strong confinements, in the presence of nanoparticles (NPs) at NP volume fractions *ϕ* up to 60%. We show that the behavior of the apparent tube diameter *d*_app_ in such nanocomposites can be greatly different from nanocomposites with nonattractive interactions. We find that this effect originates, based on a mean field argument, from the geometric confinement length *d*_geo_ at strong confinement (large *ϕ*) and not from the bound polymer layer on NPs (interparticle distance ID <2*R*_g_) as proposed recently based on experimental measurements. Close to the NP surface, the entangled polymer mobility is reduced in attractive nanocomposites but still faster than the NP mobility for volume fractions beyond 20%. Furthermore, entangled polymer dynamics is hindered dramatically by the strong confinement created by NPs. For the first time using simulations, we show that the entangled polymer conformation, characterized by the polymer radius of gyration *R*_g_ and form factor, remains basically unperturbed by the presence of NPs up to the highest volume fractions studied, in agreement with various experiments on attractive nanocomposites. As a side-result we demonstrate that the loose concept of ID can be made a microscopically well defined quantity using the mean pore size of the NP arrangement.

## Abstract 2

Conformations, entanglements, and dynamics in attractive polymer nanocomposites are investigated by means of coarse-grained molecular dynamics simulation, for both weak and strong confinements, at nanoparticle volume fractions *ϕ* up to 60%.

## Graphical abstract

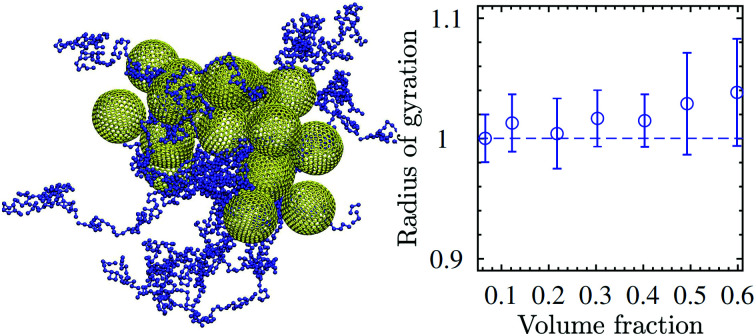

## 1 Introduction

The proper dispersion of nanofillers in a polymer matrix is a prerequisite in the diverse attempts to improve the properties of a base polymer material.^1^ One possible way to achieve a good dispersion and distribution of nanoparticles (NPs) is to make use of polymers and NPs that are mutually attractive. Attractive polymer nanocomposites are often identified by the effective attractive interaction between polymer matrix and NPs which results in the miscibility and homogeneous dispersion of NPs within the polymer matrix. The dispersion state of the NPs and hence the effective attraction can be expressed in terms of the polymer–NP Flory–Huggins interaction parameter *χ*_p–NP_, that is required to be *χ*_p–NP_ < 0.5 to avoid (micro)phase separation. This interaction parameter *χ*_p–NP_ is available from experimental measurements of the mixing free energy as well as molecular dynamics simulations utilizing thermodynamic integration.^2^ For nanocomposites containing carbon nanotubes, *χ*_p–NP_ had been calculated from the square of pure-component solubility parameters for many different polymers.^3^ An effective attraction^4^ can originate either from hydrogen bonds,^5–20^ π–π stacking^21–24^ and ionic^25–32^ or other types of interaction.^23,33–36^ The addition of NPs alters polymer rheology,^37–41^ which affects transport^42,43^ and flow.^44–46^ Polymer nanocomposites with very high NP loadings offer a lot of applications in energy storage, or as membrane or coatings, however they are still difficult to be prepared due to poor processability.^47,48^ As the addition of NPs furthermore influences polymer dynamics (diffusion, reorientation),^49–52^ a fundamental understanding of the system's dynamics allows for the improvement and design of polymer processing conditions.^53^ Despite the progress made so far, the existing studies were not able to univocally answer important questions related to conformational and dynamical aspects within such systems.

It remained unclear whether the addition of NPs (at any amounts) to a polymer matrix alters the polymer conformations, although this issue has been addressed in several previous, mostly experimental, works.^17,33,54–69^ Knowledge about the polymer conformation is essential to conclude about the existence of internal stresses, the interconnectivity of the network of chains and NPs, and to estimate characteristic relaxation times that affect various material properties. In particular, there is evidence for unperturbed conformational chain behavior, by small angle neutron scattering (SANS), of an athermal system comprising polystyrene (PS) chains and dispersed nanosilicas.^60,70^ In those studies, the polymer radius of gyration *R*_g_ exceeded the NP radius *R*_NP_ by a factor between 1.9 and 3.9.^60^ A similar conclusion was drawn for nanocomposites exhibiting a smaller ratio (*R*_g_/*R*_NP_ = 0.98–2.13), again using SANS, containing polymers and NPs that attract each other, such as poly(methyl methacrylate) (PMMA) chains and nanosilicas^17^ or syndiotactic s-PMMA chains and polyhedral oligomeric silsesquioxane (POSS) (*R*_g_/*R*_NP_ = 10–20).^59^ Unperturbed dimensions were also found for an attractive poly(ethylene oxide) (PEO)–nanosilica composite with an even smaller ratio (*R*_g_/*R*_NP_ = 0.28),^71^ at large NP volume fractions of up to *ϕ* = 53%.^72^ In contrast, PS chains were found to expand by up to 20% in the presence of PS-crosslinked NPs (for ratios *R*_g_/*R*_NP_ = 1.6–5.7)^55^ or carbon nanotubes.^58^ A dramatic expansion of polymers by almost 60% at high loading was observed for poly(dimethyl siloxane) (PDMS) in the presence of soft polysilicate *R*_NP_ = 1 nm NPs (ratios *R*_g_/*R*_NP_ = 6–8).^56,57^

Not only the static aspects remain an open issue when NPs are added, but so do the dynamical quantities. Such include relaxation times required to predict the rheological behavior or more generally, the response to external fields, as well as diffusion rates that affect polymer processing conditions. In a series of works, Gam *et al.*^73,74^ observed a decrease in the polymer diffusion coefficient as the NP volume fraction for athermal polystyrene/silica nanocomposites increased. Moreover, the tube diameter, characterizing dynamical aspects of entangled polymer matrices, was found to increase with NP loading from neutron spin echo measurements on nanosilica/PEP composites (which can be considered as nanocomposites with nonattractive interactions).^75^ In attractive PEO–nanosilica nanocomposites, however, a different behavior on the tube diameter was observed recently by Senses *et al.*^72^ Furthermore, for a strongly attractive nanocomposite material such as poly(2-vinylpyridine) (P2VP) polymers and nanosilicas, weakly adsorbed chains eventually desorbed from the NP surface, while strongly adsorbed chains remained bound for experimental time scales available to elastic recoil recovery (ERD) and Rutherford Backscattering spectrometry (RBS).^76^ The bound-layer thickness was found up to *R*_g_/2 distance from the NP surface and was affected by the strength of attraction.^12,76–78^

Several simulation efforts have tried to explore selected static and dynamic features. Most of them have focused on either a very dilute^5,52,54,79–89^ or rather moderate NP volume fractions (*ϕ* < 25%)^54,68,90–95^ except the Monte Carlo work by Sharaf and Mark^96^ for a dense athermal system, in which the polymer chains were significantly confined between the NPs and conformations were addressed. In addition, the work by Lin *et al.*^97^ focused on the mechanical enhancement under tensile deformation for very high NP loadings. Coarse-grained models for NP/polymer mixtures have revealed that polymers tend to expand with increasing *ϕ* for relatively small NPs and certain volume fractions,^23,68,96,98^ as long as 2*R*_NP_ < *R*_g_ and that there was also an attractive interaction between NPs and polymers.^66,84,87,98,99^ This result was in agreement with the simulation effort in athermal nanocomposites,^68,91,96,100^ where it was shown that the tube diameter increased with NP loading beyond *ϕ* = 20%, whereas at low loading, it remained constant,^100^ in agreement with the above-mentioned experimental observations.^75^ Another study showed that the disentanglement of chains was enhanced when small NPs were dispersed in the polymer matrix (up to *ϕ* ≈ 27%) due to the larger confinement and expansion of chains.^90^ In addition, polymer dynamics has been studied by simulations either of short chains^82,83,86,101–104^ or at a low NP loading.^82,83,86,88^

However, there has not yet been any computational effort to address entangled polymer conformations, entanglements and dynamics simultaneously in attractive nanocomposites from low to very high NP loading (*ϕ* > 40%). The present works aims at closing this gap and developing a picture that captures both static and dynamic aspects of both the NPs and the polymers, from dilute to extreme loadings. After presenting the model and methodology (Section 2), we focus on shedding light on the (i) polymer structure, (ii) entanglements and (iii) dynamics in attractive nanocomposites up to a very high NP loading. To this end, we first explore how spherical NPs, whose diameter is to the order of, or larger than *R*_g_, affect polymer dimensions (Section 3.1), as characterized by the radius of gyration and form factor, and then compare this to experimental measurements (Section 3.2). Secondly, we calculate and evaluate polymer and NP dynamics for different NP loadings, while focusing on the bound layer which affects dynamic properties and reinforcement^105–107^ (Section 3.3). Thirdly, we investigate the entanglement network for different NP loadings in an attempt to address open questions formulated earlier by Senses *et al.*^72^ for attractive nanocomposites (Section 3.4). While Senses claimed that the bound layer is responsible for a constant *d*_app_ at high *ϕ*, we find a behavior that originated, based on a mean field assumption, wholly from the geometrical confinement.^78,108^ Conclusions are offered in Section 4.

## 2 Model and methodology

We use a coarse-grained model that is known to capture the relevant dynamics and structure of simple hybrid polymer/nanoparticle systems. Our systems are composed of spherical NPs having beads on the surface and multibead-spring linear polymer chains with *N* = 100 or *N* = 200 monomers (Kremer–Grest model^109^), where each bead represents a number of monomers.^110,111^ Adjacent beads within chains are connected by anharmonic springs, while the impenetrable NPs are modeled as rigid, mobile objects whose surfaces are covered by surface beads.^112^ We use Lennard–Jones (LJ) reduced units throughout this manuscript.

To be more specific, adjacent beads *i* and *j* separated by a spatial distance *r*_*ij*_ within polymer chains are connected using finitely extensible nonlinear elastic (FENE) springs^109,113–117^1
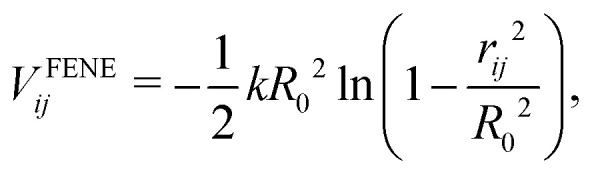
when in applying eqn (1), the maximum bond length and spring coefficient are set to *R*_0_ = 1.5 and *k* = 30, respectively, as in previous works on neutral polymers.^109,114^ All monomer or NP surface beads interact *via* a truncated, purely repulsive LJ potential *V*^LJ^_*ij*_, also known as the Weeks–Chandler–Anderson (WCA) potential, whose corresponding force acts along the line between the centers of mass of two particles.^118^ It is denoted as2
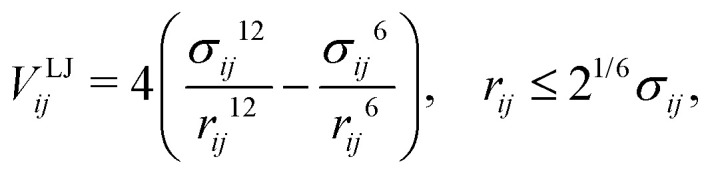
where *r*_*ij*_ represents the spatial distance between any pair of beads *i* ≠ *j*. The interaction between monomer beads and NP surface beads also contains the attractive part of the LJ potential *V*^LJ^_*ij*_ and is truncated at *r*_*ij*_ = 2.5*σ*_*ij*_. In the absence of NPs, the entanglement length^119^ of this polymer model is *N*_e_ ≈ 86, as calculated by the modified S-coil estimator.^120^ The Lorentz–Berthelot mixing^118^ rule *σ*_*ij*_ = (*σ*_*i*_ + *σ*_*j*_)/2 is used; *σ*_*i*_ = 1, if particle *i* belongs to the set of monomers, and *σ*_*i*_ = 0.4, if *i* belongs to the set of surface beads of the NPs. The modeled polymer nanocomposites consists of spherical rigid NPs with a baseline radius of 3.75 (implying an effective NP radius of *R*_NP_ = 3.75 + 0.7/2 = 4.1, obtained by adding the average monomer–NP surface bead size to the baseline radius), and are fully covered with 720 surface beads in a dense polymer melt. The mass of an NP surface bead, *m*_NP_ = 0.49, is chosen so that the NP mass density, calculated as *ρ*_NP_ = 720 × *m*_NP_/*V*_NP_ with *V*_NP_ = 4π*R*_NP_^3^/3, is ≈1.5 times the mass density *ρ* = *nNm*/*V*(1 − *ϕ*) of the polymer matrix, with monomer mass *m* = 1 (specifying the mass unit), simulation box with volume *V*, number of chains *n*, and NP volume fraction *ϕ* = *n*_NP_*V*_NP_/*V*, where *n*_NP_ denotes the number of NPs.

**Fig. 1:**
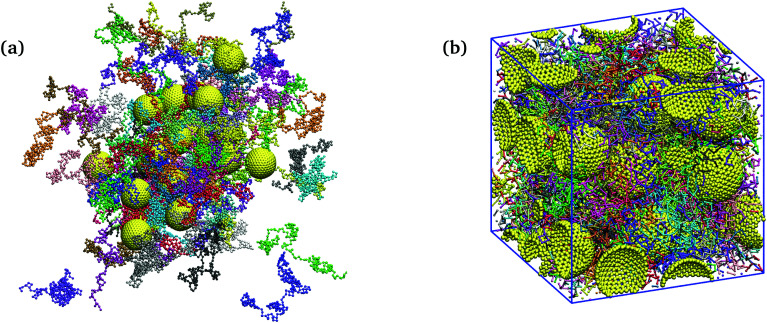
Simulation snapshots. Unwrapped (a), and wrapped (b) coordinates of an attractive polymer nanocomposite at NP volume fraction of *ϕ* = 30%, consisting of 24 NPs (golden spheres) and 72 polymer chains (colorful beads, *N* = 200 beads per chain) in a cubic simulation box with the dimensions 28.34 × 28.34 × 28.34.

A simulation snapshot of a system with NP volume fraction of *ϕ* = 30% is shown in Fig. 1. All simulations were started from random distribution of NPs configurations of nanocomposites^98^ at pressure *P* = 4.84 and temperature *T* = 1 for a duration of 5 × 10^4^ LJ time units. Subsequently, *NVT* ensemble simulations were performed, for a duration of 5 × 10^5^, at *T* = 1 by means of a Nosé–Hoover thermostat with a damping time of 0.4.^117,121^ Finally, *NVT* production runs were performed at *T* = 1 for another 5 × 10^5^ time units. Due to the choice of the FENE parameters, cutoff, and temperature, the mean bond length is *b*_0_ ≈ 1. The linear size of the simulation cell was chosen larger than the root mean square end-to-end distance of the polymer in each case. An integration time step equal to Δ*t* = 0.005 was used for polymer melts and nanocomposites. The molecular dynamics simulations were performed using the LAMMPS package.^122^

## 3 Results and discussion

### 3.1 Polymer structure and conformation

**Fig. 2:**
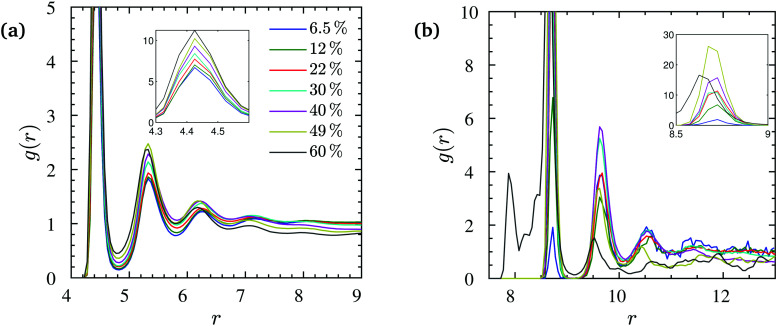
(a) NP center–monomer and (b) NP center–NP center radial distribution functions *g*(*r*) at different NP volume fractions (*N* = 200). The insets show the corresponding values of the first peaks in *g*(*r*) (or the second peak for *ϕ* = 60% in (b)).

For all nanocomposites selected for the present study, NP dispersion was achieved at all NP volume fractions *ϕ*. This is quantitatively supported by the monomer–NP center and NP center–NP center radial distribution functions (RDFs) in Fig. 2. In particular, it can be seen in Fig. 2a (for *N* = 200) that a well-defined polymer layering was formed around the NP surface. NP loading has a moderate effect on polymer–surface NP bead contacts. On one hand, upon increasing *ϕ*, the magnitude of the first peak in Fig. 2a increases, implying more polymer–surface NP bead contacts. On the other hand, it can be seen in Fig. 2a that pronounced NP–NP contacts do not exist (distance *r* = 2*R*_NP_) for *ϕ* < 60% NP volume fraction. At the highest loading (*ϕ* = 60%) there are some NP–NP contacts, but the first peak in Fig. 2b still has a lower height than the first peak in Fig. 2a denoting NP dispersion in the polymer matrix. This first peak in Fig. 2b happens at a radial distance of ≈8 that is only slightly larger than the distance between NP–NP centers in full contact calculated as 2(3.75 + 0.4/2) = 7.9. The appearance of the first peak is merely due to the occasional NP–NP contact while their preferred equilibrium position is in the radial distance corresponding to the profoundly larger second peak at *r* ≈ 9, due to the presence of a polymeric monolayer coating the NP surface. A similar behavior is observed for the NP center–NP center RDF of *N* = 100 chains, as shown in Fig. S1 of the ESI.†

**Fig. 3:**
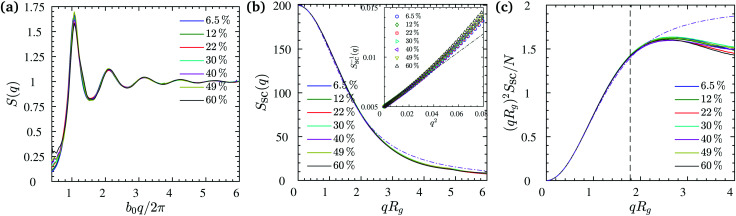
(a) Static structure factor, and (b) single chain static structure factor measured at different NP volume fractions for the systems containing chains of *N* = 200 beads each. Dash-dotted line in (b) indicates the Debye scattering function. The inset shows inverse form factor *S*_sc_^−1^ as a function of *q*^2^ at small *qR*_g_ ≪ 1 for *N* = 200 (data for *N* = 100 shown in Fig. S3, ESI†). From the initial slope the radius of gyration is determined to be the same with the value of *R*_g_ = 7.4 ± 0.1 for all NP volume fractions. (c) Kratky plots corresponding to (b). The dash-dotted line shows the Kratky-representation of the Debye function as *x*^2^*S*_sc_(*x*)/*N* = 2[exp(−*x*^2^) + *x*^2^ − 1]/*x*^2^ with *x* = *qR*_g_. The vertical dashed line marks the corresponding *q* where *qR*_NP_ = 1.

The coherent static structure factor of the polymeric subsystem, experimentally accessible *via* neutron scattering, is defined by3
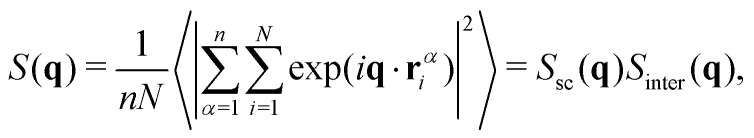
where **r**^*α*^_*i*_ is the position of the *i*th monomer of chain *α* and **q** is the wave vector; 〈⋯〉 denotes an ensemble average. *S*_sc_ is the single chain structure factor (or form factor) that describes the intramolecular correlations as4
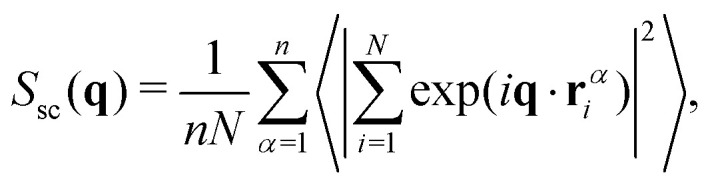
while *S*_inter_ characterizes the structure of an artificial system, where each polymer is replaced by its center of mass. Our systems are isotropic and *S*(**q**) = *S*(*q*) is radially symmetric. This static radial structure factor is depicted in Fig. 3 at different NP volume fractions for systems containing chains with *N* = 200. It can be seen that the NP loading does not affect the single chain structure factor, while it slightly changes the first peak in *S*(*q*) at *q* ≈ 2π/*b*_0_, denoting the distance between the nearest monomer neighbors. The corresponding Kratky plots – (*qR*_g_)^2^*S*_sc_/*N vs. qR*_g_ – are given in Fig. 3c, and a slight difference amongst different NP volume fractions seems to appear within the *qR*_g_ > 3 regime while the conformational statistics at length scales of the inter-NP-distance is seen to remain unaltered by *ϕ*. Apart from the regime that reflects the local stiffness our excluded volume chains, and a minor effect of NP surface on the orientational freedom of temporarily absorbed bonds, the measured form factor is captured very accurately by the Debye scattering function for random walks, *S*_sc_(*x*) = 2*N*[exp(−*x*^2^) + *x*^2^ − 1]/*x*^4^ with *x* = *qR*_g_, for all NP volume fractions, especially within the *qR*_g_ < 2.5 regime, which exceeds the Guinier regime (*qR*_g_ ≪ 1), as depicted by the dash-dotted line in Fig. 3b. Moreover, a power law behavior *S*_sc_ ∝ (*qR*_g_)^−1/*ν*^ with *ν* = 0.50 ± 0.01 is observed for the 1 ≪ *qR*_g_ ≪ *R*_g_/*b*_0_ regime, indicating that the chains behave as if they were in an ideal melt state under equilibrium conditions; see Fig. S2 (ESI†).

**Fig. 4:**
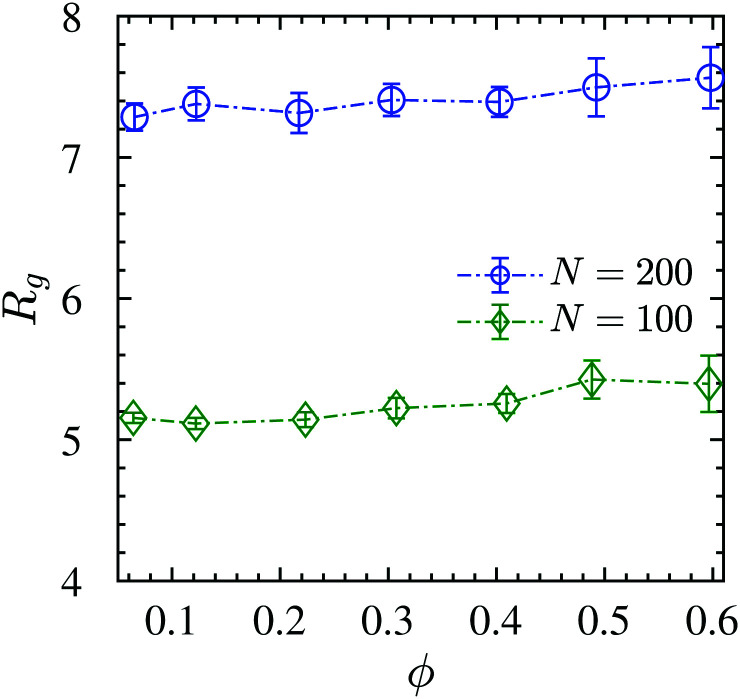
Radius of gyration *R*_g_*versus* NP volume fraction *ϕ*, obtained from the bead coordinates. Dash-dotted line is guide to the eyes.

Furthermore, the mean squared radius of gyration *R*_g_^2^ of molecules, the average squared distance between monomers and the center of mass of their molecules in a given conformation is defined as^98,111^5
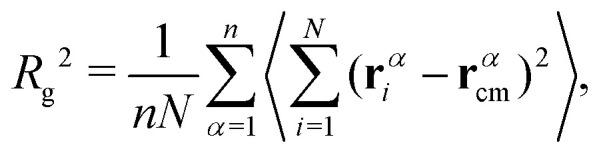
where 
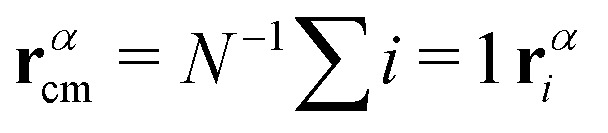
 is the instantaneous center of the mass of chain *α*. We show in Fig. 4 that, within the error margin, the overall polymer radius of gyration of *N* = 100 or *N* = 200 remain unperturbed up to a very high load (*ϕ* = 60%), which denotes a very strongly confined region. The simulation predictions are in agreement with different experimental efforts in attractive nanocomposites, such as for PMMA/nanosilica mixtures by Jouault *et al.*^17^ up to *ϕ* ≈ 30% loading. The same unperturbed behavior is observed not only in attractive PEO/nanosilica mixtures,^72^ up to *ϕ* ≈ 53%, but also in an athermal PS/nanosilica mixtures, up to *ϕ* ≈ 32% loading.^60^

We use the single chain static structure factor within the *qR*_g_ ≪ 1 regime as an alternative way to calculate the *R*_g_, as it behaves as follows, *S*_sc_(*x*) = *N*[1 − *x*^2^/3 + *O*(*x*^4^)] with the dimensionless *x* = *qR*_g_. Therefore, the radius of gyration can be evaluated from the following relation^114^*NS*_sc_^−1^(*q*) ≈1 + *q*^2^*R*_g_^2^/3 at *qR*_g_ ≪ 1 as calculated from the initial slope in the inset of Fig. 3b for *N* = 200 chains. The same value of *R*_g_ = 7.4 ± 0.1 was obtained from the form factor for all NP loadings, which is in perfect agreement with the direct measurements of Fig. 4 within the statistical error bars. This behavior also remained the same for *N* = 100 chains; see Fig. S3 (ESI†) for the single chain static structure factor and the unperturbed *R*_g_ for different NP loadings. This alternative approach further validates the observation that the *R*_g_ remains unperturbed over the relatively large NP loading range studied here. We investigated as well the tensors of gyration of all individual chains, their eigenvalues and invariants, and extracted various shape parameters. We find that there is no significant aspherity, and thus no deviation from random walk behavior, so that the radius of gyration already captures the conformational aspects (Fig. S4, ESI†).

In the following three sections we calculate, first, the degree of confinement of chains in the nanocomposite, then the polymer and NP dynamics, and finally their entanglements and corresponding effective tube diameter for different NP volume fractions and polymerization degrees.

### 3.2 Interparticle distance and pore size

**Fig. 5:**
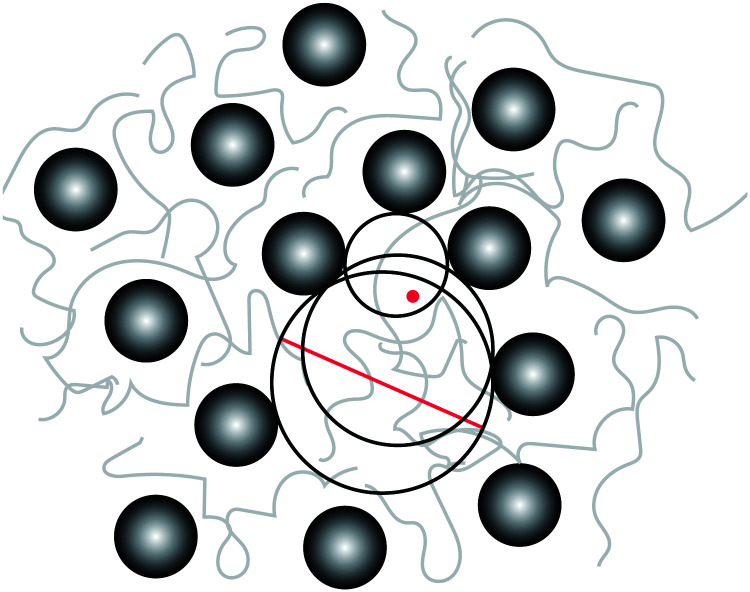
Pore size definition. At a given point (red dot) that can potentially be reached by polymers, the pore radius is defined as the radius of the largest sphere (containing that point), which can be placed without any overlap with the NPs. The diameter of this particular sphere is marked by the red line. The pore size histogram is sampled by visiting all allowed points with equal probability (algorithmic details provided in Section S4, ESI†).

**Fig. 6:**
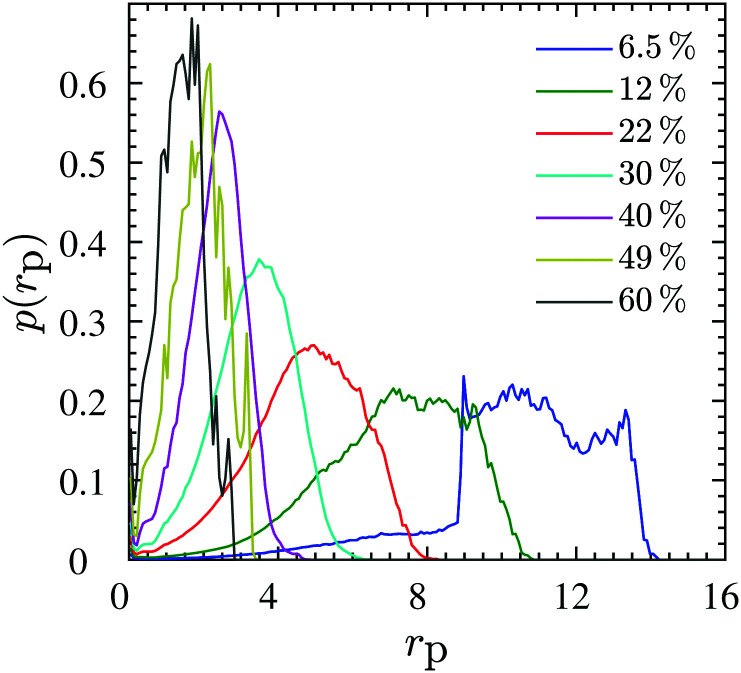
Data for *N* = 200. Pore size distribution *p*(*r*_p_) at different NP volume fractions *ϕ* mentioned in the legend, normalized such that 
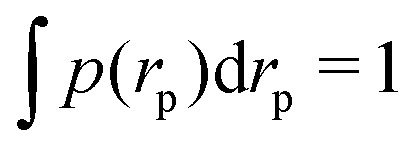
. Similar distribution plots for *N* = 100 chains are presented in the Fig. S5 (ESI†).

**Fig. 7:**
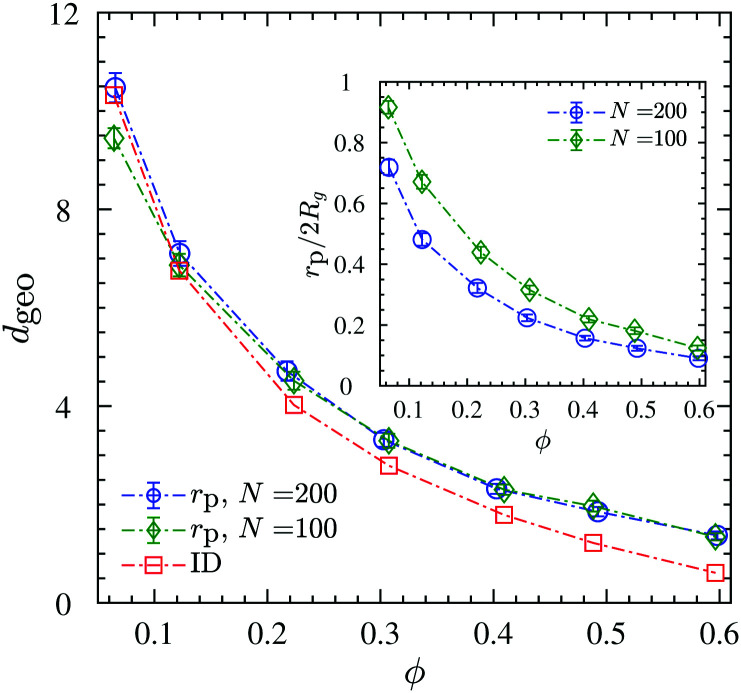
Average confinement length *d*_geo_. Mean pore size (*r*_p_), and interparticle distance (ID) between NPs with the assumption of random dense packing as a function of NP volume fraction. The inset shows the confinement ratio *d*_geo_/2*R*_g_ (identifying *d*_geo_ = *r*_p_) as a function of NP volume fraction. Dash-dotted lines are guides to the eye.

The chains in a nanocomposite experience a geometrical confinement effect imposed by the presence of the NPs. The degree of confinement is often estimated by a mean (surface–surface) interparticle (ID) distance between NPs, assuming homogeneous distribution of NPs, as^74,123^6
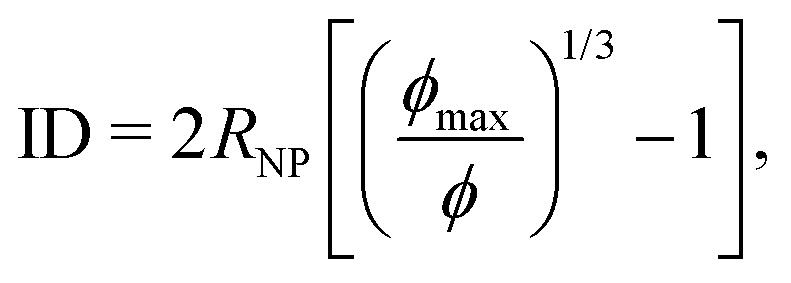
where *ϕ*_max_ is the maximum packing density of the NPs which depends on their microstructure in the system; *e.g.* for a random dense packing *ϕ*_max_ = 2/π. Because the so-defined ID is based on an assumption that we do not need to make—as we have access to the full configuration of our systems at any time—, and because there is no unique definition of an interparticle distance that could be used for a real system, we calculate the actual geometrical pore size distributions in the system as shown in Fig. 6. This distribution is constructed from the radius of the largest sphere that can be placed without any overlap with the NPs at a position that is chosen with equal probability from the space accessible by polymers, see Fig. 5 for a schematic and Section S4 (ESI†) for algorithmic details. We then compare the mean pore size against the ID estimated by eqn (6)—with the assumption of random dense packing—in Fig. 7. Upon increasing the NP loading the *p*(*r*_p_) distribution shifts to the left and becomes sharper around its mean value. Therefore, both mean pore size and its variance decrease as a result of NP loading. A similar pore size distribution trend we observe for both chain lengths studied here, see Fig. S5 (ESI†) for the *N* = 100 results. The mean geometrical confinement radius *r*_p_ obtained from the pore size analysis is consistent with the ID estimate from the formula (eqn (6)), especially at smaller NP loadings of *ϕ* < 30% as indicated in Fig. 7. We also find that *r*_p_ is independent of the chain length, and depended on the NP volume fraction for the same NP radius. We have thus replaced the ID estimate by a quantity *r*_p_ that can actually be measured by either transmission electron microscopy (TEM)^124,125^ or small angle X-ray scattering^126^ or a fluorescence method with 3D imaging technique.^127^ It gives rise to the so-called geometric confinement length *d*_geo_, for which we use *d*_geo_ = *r*_p_ (instead of *d*_geo_ = ID).

The ratio *d*_geo_/2*R*_g_ denotes the degree of confinement that polymers experience from NPs. It is depicted for *N* = 100 and *N* = 200 chains in the inset of Fig. 7 for different NP loadings. For both chain lengths studied here, it holds that *d*_geo_/2*R*_g_ < 1, hence, they are in a strong geometrical confined regime, and the confinement ratio decreases with increasing NP loading.

### 3.3 Polymer dynamics

**Fig. 8:**
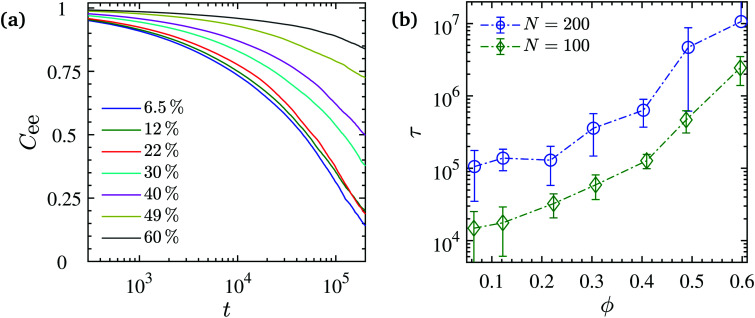
(a) Chain end-to-end vector autocorrelation function *C*_ee_(*t*) at various NP volume fractions for *N* = 200. In every case *C*_ee_(0) = 1. (b) Average chain end-to-end relaxation time *τ* obtained from fitting of the stretched exponential function to the *C*_ee_(*t*) numerical values (corresponding stretched exponents *β* < 1 in Fig. S8, ESI†). Dash-dotted lines are guides to the eye.

In this section we focus our attention on polymer rotational- and translational dynamics and its spatial modulation in the neighborhood of NPs. To calculate the chain's orientational relaxation time, we measured the autocorrelation function *C*_ee_(*t*) of the chain end-to-end vector **R**_ee_ = **r**_*N*_ − **r**_1_ defined by7
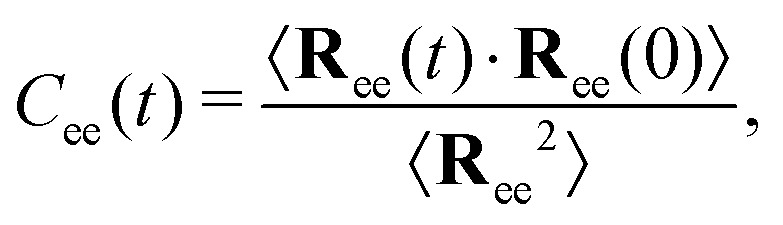
averaged over all chains, and all times. Fig. 8a shows *C*_ee_ at different NP volume fractions. It indicates that up to *ϕ* ≈ 22%, the average chain orientational relaxation is rather insensitive to the presence of the NPs, while for higher NP loadings, the chain relaxation slows down. In order to quantify this, we calculated the average end-to-end relaxation time by fitting a stretched exponential function to the numerical results of *C*_ee_(*t*) as shown by

8 *C*_ee_(*t*) = exp[−(*t*/*τ*_r_)^*β*^],

and obtain the average orientational relaxation time *τ* as9
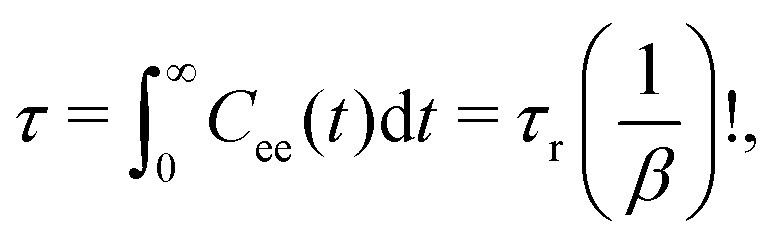
where *x*! = *Γ*(*x* + 1) denotes the generalized factorial or shifted gamma function. The average orientational relaxation time *τ* is shown in Fig. 8b as a function of the NP volume fraction. See the corresponding stretched exponents *β* in Fig. S8 (ESI†). The relaxation time is constant up to *ϕ* ≈ 22% and then increases roughly by two orders of magnitude at an NP loading of *ϕ* = 60%. This increase is in agreement with a previous simulation study of nanocomposites.^128^

**Fig. 9:**
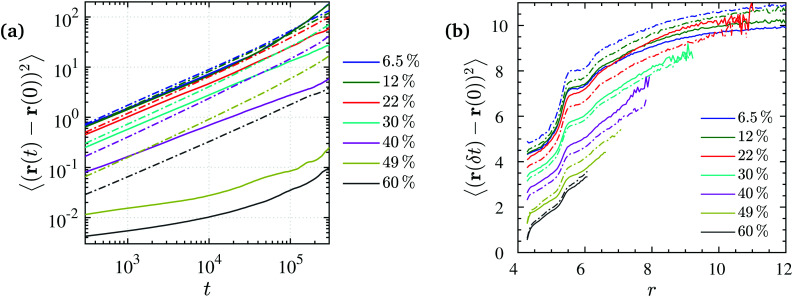
(a) Mean-squared displacement (MSD) of NPs (solid lines) and polymer COMs (dash-dotted lines) at different NP volume fractions for *N* = 200. Both NPs and chain COMs indicate sub-diffusive behavior over the range of volume fractions studied here. (b) Short-time MSD of monomers relative to their closest NP center, measured at δ*t* = 250 for *N* = 200 (solid lines) and *N* = 100 (dash-dotted lines) chains. MSD is measured as a function of monomer (initial) radial distance relative to its closest NP center at different NP volume fractions.

The mobility of polymers and NPs is very differently affected by the NP volume fraction. Regarding the overall translational mobility, we calculated the mean square displacement of polymers center of mass and NPs for different NP loadings. A sub-diffusive behavior is observed for both NPs and chain COMs over the entire NP volume fraction range studied here as shown in Fig. 9a likely due to the chain entanglements and NP confinements which may result in a cooperative dynamics. Such behavior has been observed not only experimentally, for NPs in a polymer matrix,^129–131^ but also from computer simulations, for polymers within porous^132^ or confined media.^133^ We can see in Fig. 9a that polymer and NP dynamics are similar at low NP volume fractions (*ϕ* = 6.5% and 12%) for *N* = 200. For higher NP loading, polymer dynamics is faster than NP dynamics, and the discrepancy increases with the NP volume fraction. A faster dynamics but with a similar trend was also observed for the *N* = 100 chains, but in that case polymer dynamics was faster than NP dynamics for any NP volume fraction, as shown in Fig. S7 (ESI†). There is a tendency for polymers to diffuse faster than NPs which gets enhanced with NP loading, not only because of their lower density relative to the NPs, but also because of the NP packing.

We note here that at the large volume fraction regime *ϕ* ≥ 49% and in particular for the entangled systems with *N* = 200, the slow chain relaxation and even slower NP mobility point to the fact that these systems represent a “solid-like” behavior. In order to ensure the sampling of equilibrium states, we started the simulations from a clustered NP state and measured the 〈*R*_g_〉 time series as well as the NP–NP pair correlation functions and found out that there is no drift in these quantities over the sampling period (see Fig. S9 and S10 (ESI†).

The short-time MSD of monomers is spatially dependent. It is shown as a function of radial distance from their closest NP in Fig. 9b for different NP loadings. The mobility of monomers increases according to their distance from NP centers, and reaches a plateau at distances far beyond the NPs surface (*r >* 9) for small volume fractions of *ϕ* ≤ 22%. This radial distance is comparable to the *R*_NP_ + *R*_g_/2 value where *R*_g_/2 is a typical thickness of the polymer bound layer observed experimentally. We further observe a somewhat smaller plateau up to the distance of *r* ≈ 5.5 that corresponds to the second solvation shell of the monomers attracted to the NP surface. Larger radial distances to the NP surface cannot be reached for the high *ϕ* because it exceeds the ID and pore radius. It appears that within the volume fraction regime *ϕ* ≤ 22% the monomer mobility is rather insensitive to the NP volume fraction, while at higher NP loadings (*ϕ* > 22%) it slows down almost linearly with increasing *ϕ*, due to NP confinement that hinders polymer motion, as well as longer lasting monomer–NP temporary contacts formed in higher NP loadings.

### 3.4 Entanglements and tube diameter

In nanocomposites, polymer/NP (topological) entanglements control the mechanical response and viscosity of the nanocomposite. According to Schneider *et al.*,^75^ a mean field relation exists between three characteristic diameters: the apparent tube diameter *d*_app_ that can be measured by neutron spin echo (NSE) experiments, the geometric confinement length *d*_geo_ already introduced, and the diameter *d*_tube_ of a “tube” in which polymer chain motion is constrained, imposed by the topological constraints of the neighboring polymer chains. The mean field equation^75^10
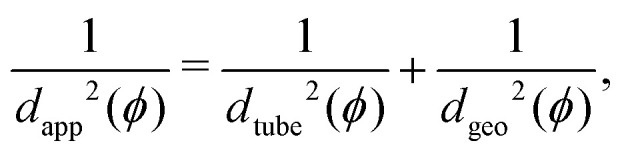
implies that the apparent tube diameter is dominated by, and always smaller than the smaller of the two other diameters.

**Fig. 10:**
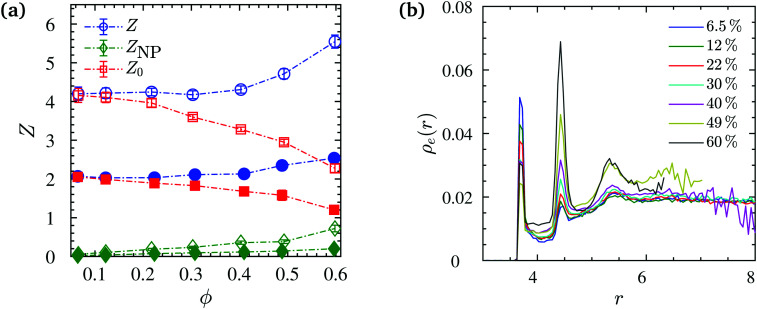
Data for *N* = 200 (open symbols) and *N* = 100 (filled symbols). (a) Mean number of polymer–polymer *Z*, and polymer–NP *Z*_NP_ entanglements per chain in the frozen NP limit as a function of NP volume fraction *ϕ*. *Z*_0_ curve shows mean number of polymer–polymer entanglements in the phantom NP limit where the NPs are simply ignored in the analysis. (b) Number density profiles of entanglements *ρ*_e_(*r*) (frozen limit) at distance *r* measured from NP center at different NP volume fractions for *N* = 200. Dash-dotted lines are guides to the eye.

In our work, we calculate the primitive path networks of polymers for all NP loadings, from which we obtained the number of “kinks” *Z* considered to be proportional to the number of entanglements per chain, *N*_e_ = *N*/*Z*. In line with previous works,^90,135^ we undertook this analysis with two limits: the phantom limit, where NPs were simply ignored (*Z*_0_), and the frozen limit, where NPs served as obstacles but did not move during the minimization procedure. In the latter case we distinguish between polymer–polymer *Z* and polymer–NP entanglements *Z*_NP_. More specifically, in the frozen limit, not only polymers but also the NPs can give rise to kinks of the shortest disconnected path. Such kinks are located on the surfaces of the NPs and denoted as polymer–NP entanglements. All the above quantities can be seen in Fig. 10a, and in particular a strong disentanglement of chains beyond *ϕ* = 20% loading in the phantom limit. The decrease of entanglements with *ϕ* in the phantom limit is due to the diminishment of the relative amount of NP obstacles that increases with *ϕ*, and qualitatively similar to chain disentanglement near flat surfaces,^136^ that do not allow to distinguish between frozen and phantom limits. In the frozen limit however, polymer–polymer and polymer–NP entanglements increase per chain with the NP loading as the amount of obstacles provided by the NP surfaces is proportional to *ϕ*.

The entanglement number density profile *ρ*_e_(*r*) is shown (Fig. 10b) as a function of distance from NP centers, as well as the rheologically relevant entanglement bulk density obtained as *ρ̄*_e_ = (*Z* + *Z*_NP_)*n*/*V*(1 − *ϕ*) (Fig. S6, ESI†). An increase in entanglement number density *ρ̄*_e_ is found for both *N* = 100, 200 chains with NP loading. We determine *ρ*_e_(*r*) using entanglements and spherical shell volumes residing within the Voronoi volume of their nearest NP. It can be seen in Fig. 10b that there is an interfacial region around NPs where the entanglement density is different from that in the bulk phase. In particular, this profile indicates two pronounced peaks within one monomer distance from the NP surface, and a minor third peak further away. The second peak (polymer–polymer entanglements) grows to the expense of the first peak (NP–polymer entanglements) with increasing *ϕ* while the *ρ*_e_(*r*) for the phantom case does not exhibit such pronounced peaks. For volume fractions *ϕ* ≥ 22%, the magnitude of the second peak in *ρ*_e_(*r*) exceeds the bulk value, and the NP surface seems to directly (NP contact) or indirectly (2nd layer) dominate the entanglement behavior.

**Fig. 11:**
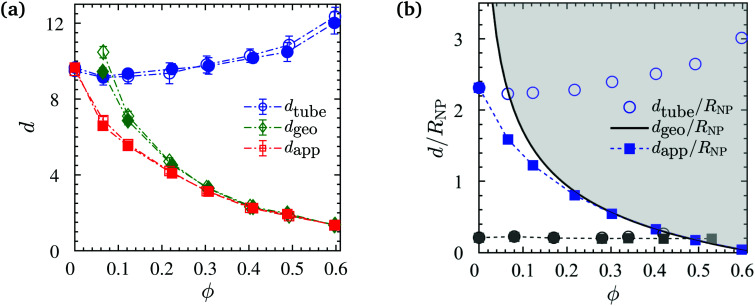
(a) Apparent tube diameter (*d*_app_) as described in eqn (10), tube diameter from primitive path analysis (*d*_tube_) in the phantom NP limit, and average confinement length from pore size analysis (*d*_geo_) as a function of NP volume fraction. Tube diameters at *ϕ* = 0 are obtained from analytical fast-converging estimators.^134^ (b) A comparison between different ratios of *d*/*R*_NP_ from the current study (blue symbols) for *N* = 200 chains and the experimental values (gray symbols) reported by Senses *et al.*^72^ for PEO/nanosilica composites (*d*_tube_ of PEO was calculated using eqn (10)). The solid black line shows the analytical formula of ID/*R*_NP_ obtained from eqn (6). The shaded gray area marks the region unattainable to the apparent tube diameter *d*_app_ (filled symbols). Dash-dotted lines are guides to the eye.

We then evaluate the tube diameter *d*_tube_ directly from the primitive path analysis as 
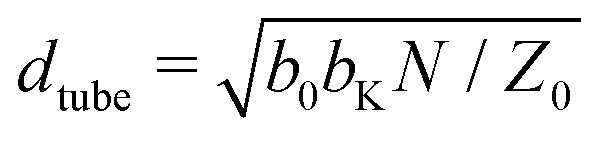
 with average bond length *b*_0_ = 0.97, Kuhn length *b*_K_ = 1.79, and the number of entanglements *Z*_0_ per chain in the phantom NP limit.^110,120,137^ As depicted in Fig. 11a (with blue symbols), it is insensitive to *N*, because both chain lengths are located in the entangled regime, and increases with increasing *ϕ*.

Upon making use of *d*_geo_ we calculate *d*_app_ from *d*_tube_*via*eqn (10). It can be seen in Fig. 11a that the apparent tube diameter decreases abruptly with the NP volume fraction up to *ϕ* = 20%. Such behavior was also observed in experimental measurements in PEO/nanosilica composites.^72^ However, this behavior was different to that observed in nanocomposites containing nonattractive interaction between polymers and NPs,^75^ where *d*_app_ remained constant up to *ϕ* = 20%. Beyond that volume fraction, *d*_geo_ coincided with *d*_app_ (red and green lines coincide for *ϕ* > 30%) as depicted in Fig. 11a, denoting that the term *d*_geo_^−2^ ≫ *d*_tube_^−2^, and thus the value of *d*_app_ depend on geometric confinement (*d*_geo_), and the disentanglement of chains originated from the random packing of NPs and the confinement they created.^50^ Since the radius of gyration is unperturbed by NP loading, it does not promote the disentanglement that was observed in previous studies.^90,100^ Moreover, the disentanglement of chains is smaller than that observed in nanocomposites with nonattractive interactions, due to the lack of expansion of the polymer radius of gyration. Again, this behavior is different from that seen in nanocomposites with nonattractive interactions,^75^ where *d*_app_ and *d*_geo_ coincide only at a very high NP volume fraction (*ϕ* > 50%).^75^

In recent quasielastic neutron scattering measurement (QENS) experiments in attractive nanocomposites, *d*_app_ has been measured by Senses *et al.*^72^ It is worth noting that in their study *R*_g_ (7 nm) ≪ *R*_NP_ (25 nm), we thus cannot directly compare with their results as *R*_g_ ≈ 2*R*_NP_ in the present work. At a given NP volume fraction their ID is 8 times larger than our ID, relative to the size of the polymers. In experiments by Senses *et al.*^72^*d*_geo_^−2^ ≈ *d*_tube_^−2^, the geometric confinement was much weaker compared with our present simulations (ID of experiments was much larger than the ID of our simulations, thus had a larger *d*_geo_), since large nanosilicas of 50 nm diameter were used. This led to a different trend that observed in experimental *d*_app_ (*d*_app_ remained constant for *ϕ* ≥ 30%).^72^ Senses *et al.*^72^ used ID/2*R*_g_ < 1 as a necessary and sufficient condition for *d*_app_ to be unaffected by *ϕ*.

Based on the mean field equation, we have thus demonstrated that this condition does not hold. To support this further, we normalized the diameters *d*_app_, *d*_tube_ (for both experiments and simulations) and ID (*d*_geo_) with *R*_NP_, for all volume fractions, and depict these normalized ratios in Fig. 11b. It can be seen that the trend of Senses *et al.*^72^ data is much more subtle than our simulation data, due to the smaller *d*_tube_/*R*_NP_ ratio. The *d*_geo_/*R*_NP_ line defines the boundary region, if approached, *d*_app_ follows *d*_geo_. The *d*_app_ of Senses apparently just reaches the *d*_geo_/*R*_NP_ line at the highest realized NP loadings. A subsequent decrease of *d*_app_ could thus not be confirmed experimentally with the NP volume fractions that were available for their investigation.^72^

## 4 Conclusions

There is still opposing experimental (and also simulation) evidence concerning entangled polymer structure and dynamics in nanocomposites. That is especially true for the technologically relevant regime of large NP volume fractions. Thus, we investigated polymer conformations, entanglements and dynamics in attractive polymer nanocomposites, up to approximately *ϕ* = 60% loading, using a coarse-grained model for NPs and polymers, by means of molecular dynamics simulations. We observe an unperturbed behavior of entangled polymer chains, for the first time using simulations (polymers exhibit ideal chain statistics for 1.27 < *R*_g_/*R*_NP_ < 1.8), even at such high NP loading, in agreement with SANS experiments of attractive nanocomposites. At relatively high NP loading (*ϕ* ≥ 30%), chains disentangle due to geometric confinement, however, chain disentanglement was not as abrupt as in nanocomposites with nonattractive interactions. In addition, we showed based on a mean field equation, that the behavior of *d*_app_ originates from the geometrical confinement length *d*_geo_ (for *ϕ* ≥ 30%) and not from the dynamics of the bound polymer layer between NPs. The effect of NP volume fraction on the *d*_app_ for nanocomposites that are not studied here, with a smaller or larger ratio between tube diameter and NP size, we expect to follow the trend observed here, and apparently supported by experiment: a moderate increase of *d*_tube_ with *ϕ* up to some critical *ϕ*, in the neighborhood of the NP volume fraction where *d*_app_ and *d*_geo_ meet. In order to explore smaller ratios of *d*_tube_/*R*_NP_, it would require simulating either stiffer polymers (where *d*_tube_ would be smaller) or nanocomposites with larger NPs (which would require larger system sizes). Entangled polymer dynamics is reduced close to the NP surface, due to the attractive interaction, and is hindered dramatically, at high NP loadings, due to confinement.

## Conflicts of interest

There are no conflicts to declare.

## Supplementary Material

SM-017-D1SM00683E-s001
