# Supplementary material for: Insights from modeling into structure, entanglements, and dynamics in attractive polymer nanocomposites
Source: Soft Matter. 2021 Jun 9;17(26):6362–73. doi: 10.1039/d1sm00683e (PMC8262555; doi:10.1039/d1sm00683e)
Supplement: SM-017-D1SM00683E-s001 [file SM-017-D1SM00683E-s001.pdf]

Electronic Supplementary Material (ESI) for Soft Matter.
This journal is © The Royal Society of Chemistry 2021

Supplementary Information: Insights from modeling into structure, entanglements, and dynamics in attractive polymer nanocomposites

Ahmad Moghimikheirabadi,[*,†] Martin Kröger,[*,†] and Argyrios V. Karatrantos[*,‡]

†*Polymer Physics, Department of Materials, ETH Zurich, Leopold-Ruzicka-Weg 4, CH-8093 Zurich, Switzerland*
‡*Materials Research and Technology, Luxembourg Institute of Science and Technology, 5, Avenue des Hauts-Fourneaux, L-4362 Esch-sur-Alzette, Luxembourg*

E-mail: ahmadm@mat.ethz.ch; mk@mat.ethz.ch; argyrios.karatrantos@list.lu

Contents

1 NP–NP radial distribution function 2
2 Single chain structure factor 2
3 Gyration tensor analysis 3
4 Pore size distribution 3
5 Entanglements 4
6 Polymer and NP dynamics 4
7 Orientational relaxation 4
8 Equilibration at large volume fractions 5
References 5

1 NP–NP radial distribution function

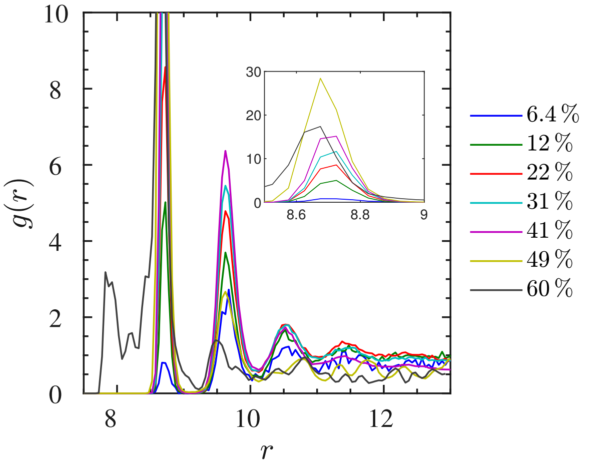

Figure S1: Data for $N = 100$. NP center–NP center radial distribution functions $g(r)$ at different NP volume fractions ϕ mentioned in the legend.

2 Single chain structure factor

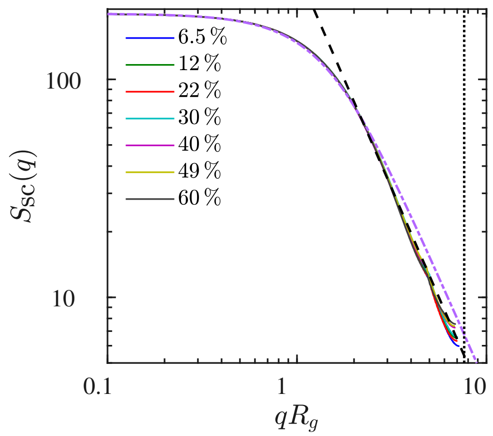

Figure S2: Data for $N = 200$. Single-chain structure factor in double-logarithmic representation. The dash-dotted line shows the Debye function, the dashed line shows the power law behavior $(qR_g)^{-1/\nu}$ with $\nu = 0.50 \pm 0.01$ obtained from fitting for the $1 \ll qR_g \ll R_g/b_0$ regime, and the dotted vertical line marks the $R_g/b_0 \approx 7.63$ value with $R_g = 7.4$ and bond length $b_0 = 0.97$.

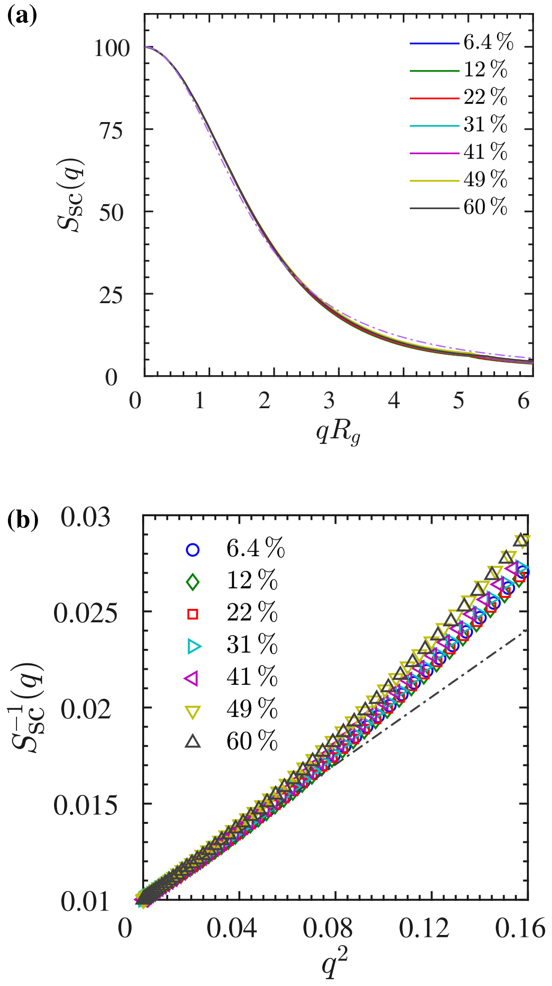

Figure S3: Data shown for $N = 100$. (**a**) Single chain static structure factor measured at different NP volume fractions for the systems containing chains of $N = 100$ beads each. (**b**) Corresponding inverse form factor $S_{\rm sc}^{-1}$ as a function of q^2 at small $qR_g \ll 1$. From the initial slope the radius of gyration is determined to be the same with the value of $R_g = 5.2 \pm 0.1$ for all NP volume fractions.

3 Gyration tensor analysis

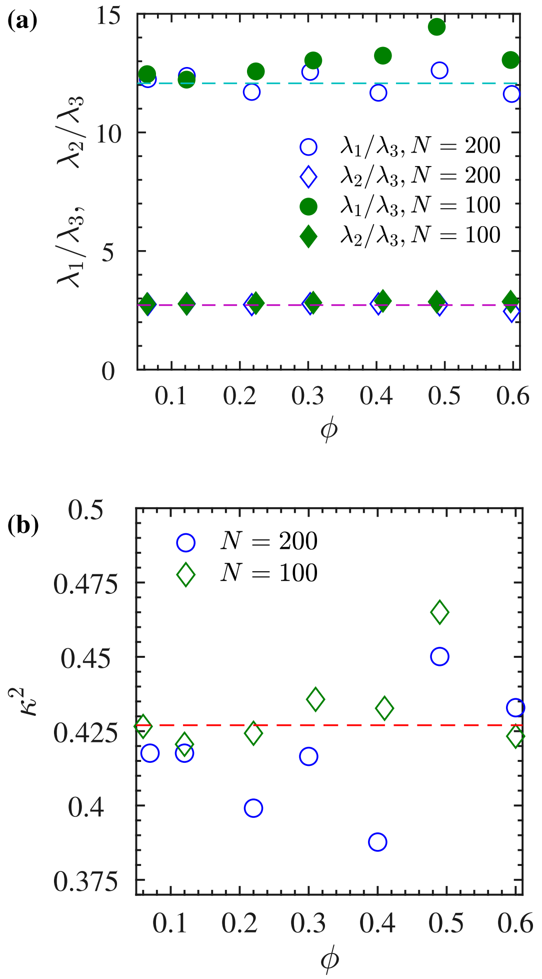

Figure S4: Gyration tensor analysis. (**a**) The ratios $\langle\lambda_1\rangle / \langle\lambda_3\rangle$ and $\langle\lambda_2\rangle / \langle\lambda_3\rangle$ for both $N = 100$, 200 chains as a function of NP volume fraction where the three eigenvalues of gyration tensor λ_1, λ_2, and λ_3 are sorted such that $\lambda_1 \geq \lambda_2 \geq \lambda_3$. The dashed lines indicate the corresponding values of $\langle\lambda_1\rangle / \langle\lambda_3\rangle \approx 12.07$ and $\langle\lambda_2\rangle / \langle\lambda_3\rangle \approx 2.72$ for the Gaussian chains.[1] (**b**) The relative shape anisotropy is defined as[2] $\kappa^2 = 1 - 3\langle I_2\rangle/\langle I_1\rangle^2$, where I_1 and I_2 are the first and second tensor invariants of the gyration tensor, $I_1 = \lambda_1 + \lambda_2 + \lambda_3$ and $I_2 = \lambda_1\lambda_2 + \lambda_1\lambda_3 + \lambda_2\lambda_3$, which do not depend on the ordering. The dashed line corresponds to the value of $\kappa^2 \approx 0.427$ if the eigenvalues were statistically independent, using the above ratios for Gaussian chains.

4 Pore size distribution

Here we provide full details about the simplest algorithm that can be used to determine the pore radius distribution $p(r_\mathrm{p})$. As mentioned in the caption of Fig. 5 at a given point in space that can potentially be reached by polymers, the pore radius r_p is defined as the radius of the largest sphere (containing that point), which can be placed into the system without any overlap with the NPs. Points that can potentially be reached by polymers are all those whose minimum distance to any of the NPs is larger than the NP radius R_NP. The pore size histogram is sampled from the pore radii by visiting all allowed points, i.e., all points outside the NP bodies, with equal probability. To ensure equal probability, a point $\mathbf{p}$ inside the simulation box is randomly created using three equally distributed random numbers, and rejected if its minimum distance to a NP is below R_NP, otherwise it is used. A useful prerequisite to find the pore radius at an accepted point in space is to once create an Euclidean distance map (EDM) on a fine regular grid with a grid constant of $\delta r = 0.05$ (this value determines the resolution of the pore radius distribution). At startup, all nodes of the EDM receive the value infinity. To construct the Euclidean distance map we loop over all NPs. For each of the NPs, say NPi, whose center is residing at position $\mathbf{R}_i$, we loop over all nodes $\mathbf{r}$ of the Euclidean map and set $\mathrm{EDM}(\mathbf{r}) = \min[|\mathbf{R}_i - \mathbf{r}|, \mathrm{EDM}(\mathbf{r})]$. With the EDM plus a random point $\mathbf{p}$ at hand, we know the EDM value $\mathrm{EDM}(\mathbf{p})$. The radius of the largest sphere that can be placed into the system without NP overlap and containing $\mathbf{p}$ must be at least $\mathrm{EDM}(\mathbf{p})$, because $\mathrm{EDM}(\mathbf{p})$ is the radius of the largest sphere that can be placed with its center located at $\mathbf{p}$. To find the pore radius at $\mathbf{p}$ we visit all those nodes $\mathbf{r}$ of the EDM map that fulfill the condition $|\mathbf{r} - \mathbf{p}| \leq \mathrm{EDM}(\mathbf{r})$. The pore radius r_p at point $\mathbf{p}$ is the largest of the $\mathrm{EDM}(\mathbf{r})$ values fulfilling this condition. We repeat the procedure for 10^6 randomly chosen points $\mathbf{p}$, giving rise to 10^6 pore radii r_p that enter the pore radius distribution.

What we described so far is a simple algorithm that can be implemented directly. We actually used a more efficient implementation, using neighbor lists, sort algorithms etc. which produces exactly the same pore size distribution, but is more difficult to be summarize in a single paragraph. As it also only differs in the speed of execution, this detail should be irrelevant for the understanding of the presented results.

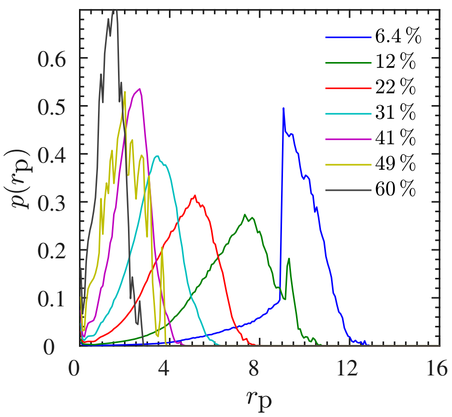

Figure S5: Data for $N = 100$. Pore size distribution $p(r_\mathrm{p})$ at different NP volume fractions ϕ mentioned in the legend, normalized such that $\int p(r_\mathrm{p}) dr_\mathrm{p} = 1$.

5 Entanglements

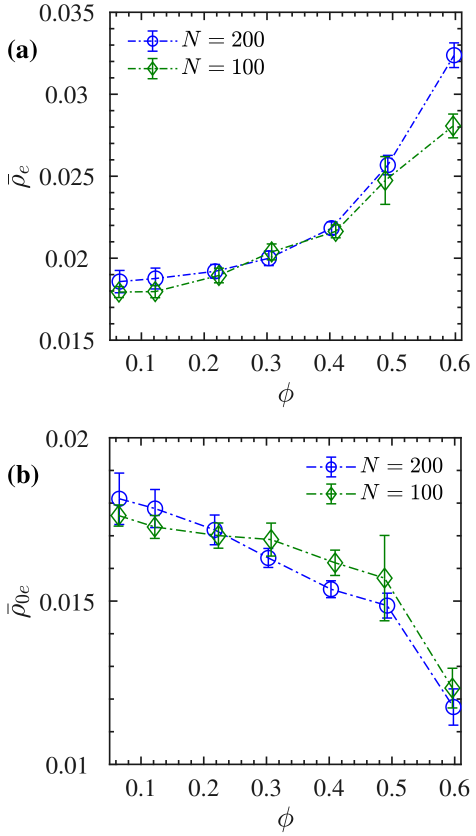

Figure S6: The average entanglement number density in the system, obtained from $\bar{\rho}_e = (Z + Z_{\rm NP})n/V(1-\phi)$ (**a**) in the frozen- and $\bar{\rho}_{0e} = Z_0 n/V(1-\phi)$ (**b**) in the phantom limit as a function of NP volume fraction for both $N = 100$ and $N = 200$ chains.

6 Polymer and NP dynamics

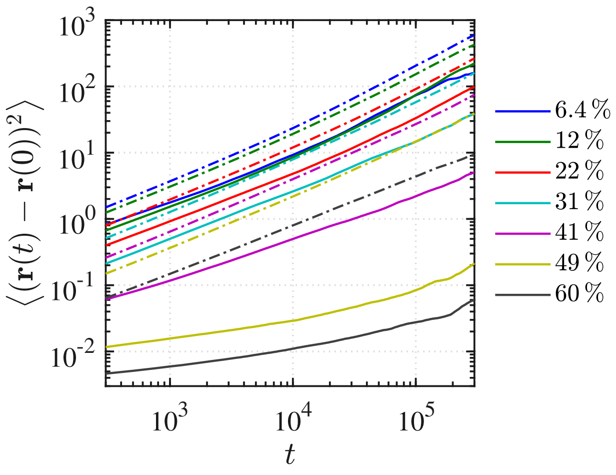

Figure S7: Data for $N = 100$. (**a**) MSD of NPs (solid lines) and polymer COMs (dash-dotted lines) at different NP volume fractions.

7 Orientational relaxation

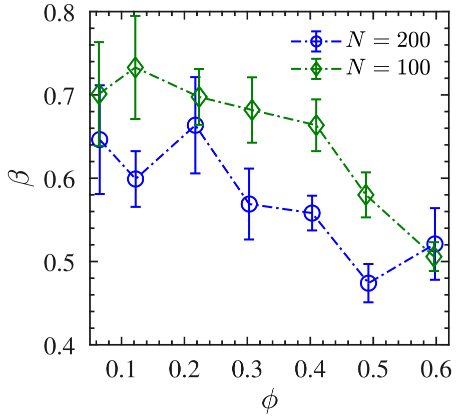

Figure S8: Stretched exponent β obtained from fitting of the KWW stretched exponential function to the $C_{\rm ee}(t)$ simulation data.

8 Equilibration at large volume fractions

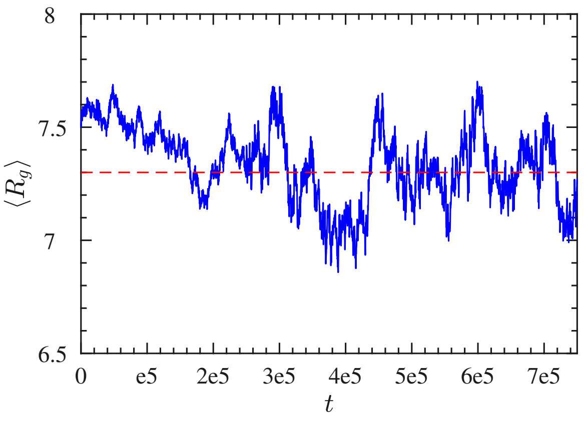

Figure S9: Data for $N = 200$. Rg time series measured for the system with a (large) NP volume fraction of $\phi = 49\%$, starting from a phase separated configuration (NPs clustered) at $t = 0$. Dashed horizontal line marks the average radius of gyration.

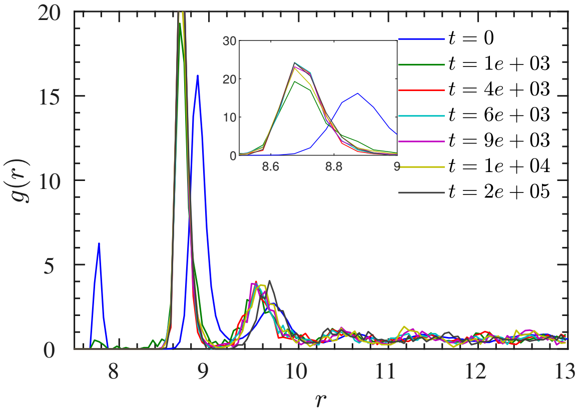

Figure S10: Data for $N = 200$. NP center–NP center radial distribution functions $g(r)$ at a NP volume fractions of $\phi = 49\%$ measured over time, starting from a phase separated configuration (NPs clustered) at $t = 0$.

References

(1) Janszen, H. W. H. M.; Tervoort, T. A.; Cifra, P. Bimodality in the spatial segment density distribution of Gaussian chains. *Macromolecules* **1996**, *29*, 5678–5687.

(2) Arkin, H.; Janke, W. Gyration tensor based analysis of the shapes of polymer chains in an attractive spherical cage. *J. Chem. Phys.* **2013**, *138*, 054904.
